# Severe Infection With Avian Influenza A Virus is Associated With Delayed Immune Recovery in Survivors

**DOI:** 10.1097/MD.0000000000002606

**Published:** 2016-02-08

**Authors:** Jianing Chen, Guangying Cui, Chong Lu, Yulong Ding, Hainv Gao, Yixin Zhu, Yingfeng Wei, Lin Wang, Toshimitsu Uede, Lanjuan Li, Hongyan Diao

**Affiliations:** From the State Key Laboratory for Diagnosis and Treatment of Infectious Diseases, Collaborative Innovation Center for Diagnosis and Treatment of Infectious Diseases, The First Affiliated Hospital, College of Medicine, Zhejiang University, Hangzhou, China (JC, GC, CL, YD, HG, YZ, YW, LW, LL, HD); and Molecular Immunology, Institute for Genetic Medicine, Hokkaido University, Sapporo, Japan (TU).

## Abstract

Human infection with avian influenza A virus (H7N9) is a concern because of the mortality rate. Previously, we characterized immunological responses during active infection with it and reported evidence of impaired antigen-presenting capability, particularly in severely affected individuals. Here we describe an investigation of immunological responses during a 1-year follow-up of survivors of H7N9 infection. Survivors of H7N9 infection were classified as having had mild (n = 42) or severe infection (n = 26). Their immune status, including human leukocyte antigen-DR expression on monocytes, and their ability to mount cytokine responses were assessed at 1, 3, and 12 months postinfection.

The total lymphocyte count and the percentages of different types of lymphocytes had normalized by 1 month postinfection. However, there was evidence of ongoing impairment of immune responses in those who had had severe infection. This included reduced human leukocyte antigen-DR expression on CD14^+^ monocytes, reduced interferon-γ production by T cells, and higher plasma levels of the matrix metalloproteinases 2, 3, and 9. By 3 months postinfection, these had all normalized.

After severe H7N9 infection, recovery of the antigen-presenting capability of monocytes and T-cell responses are delayed. This may lead to an increased vulnerability to secondary bacterial infections.

## Abstract 2

Supplemental Digital Content is available in the text

## INTRODUCTION

Avian influenza A (H7N9) is a subtype of the influenza viruses and was first detected in March 2013 in Shanghai, China. Since then, more than 560 confirmed cases of human infection with H7N9 virus, including 204 deaths, have been reported.^1^ The high case fatality rate is a particular concern. Poultry has been identified as the main source of human infection, either as a consequence of exposure to live poultry or an environment contaminated by poultry.^2–4^ After the initial wave of infections in spring 2013,^5,6^ a second occurred in the south of China the following winter.^7,8^ Subsequently, further infections occurred in the fall of 2014, as a more typical seasonal pattern,^9,10^ which were still going on.

Patients with H7N9 infection are often lymphopenic,^11–13^ and a striking feature of the infection is the high rate of serious complications, including acute respiratory distress syndrome (ARDS)^12^ and bacterial infections,^14^ causing severe pneumonia, multiple organ failure, and sometimes death.^15,16^ This prompted us to investigate the immune status of patients during and after infection with H7N9. To date, little is known about restoration of the immune system after H7N9 infection.

Previously we reported that, when compared with patients with mild H7N9 infection, those with severe infection had significantly lower levels of T lymphocytes and monocytes, though both of them were commonly lymphopenic. Moreover, the frequency of human leukocyte antigen-DR (HLA-DR) on CD14^+^ cells, a measure of their antigen-presenting capability, was negatively correlated with the severity of H7N9 infection. The reduced antigen-presenting capability might result in impairment of T-lymphocyte responses and lead to decreased interferon (IFN)-γ and interleukin (IL)-17 levels. These findings suggest that HLA-DR expression on CD14^+^ cells could be a biomarker, helping to predict the clinical course of those infected with H7N9.^17^

Currently, little is known about immune recovery and the ability of patients to respond to exogenous antigenic stimuli after mild and severe H7N9 infections. Here we report a study of the immunological recovery over a period of a year in individuals classified as having had mild and severe H7N9 infections. In particular, we investigated their antigen-presenting capabilities, inflammatory responses, and the levels of certain matrix metalloproteinases (MMPs) correlating these with the severity of the initial infection.

## METHODS

### Ethical Experimentation

The study was approved by the institutional ethics committee of the First Affiliated Hospital College of Medicine, Zhejiang University (Reference 2013–166). The methods were carried out in accordance with the approved guidelines. Written consent was obtained from the patients.

### Patients

We recruited 68 patients with confirmed H7N9 virus infection who had been hospitalized at the First Affiliated Hospital College of Medicine, Zhejiang University, China. All had presented with respiratory symptoms and unexplained chest x-ray infiltrates. The cases were divided into those who were mildly affected (n = 42) and those with severe infection (n = 26). Patients were identified as severe cases according to one or more of the criteria (during the infection phage) as we previously described.^17^ Meanwhile, the disease severity was evaluated by calculating the Acute Physiology and Chronic Health Evaluation II (APACHE-II) score^18^ of each enrolled patient. We also collected demographic and other background data on the patients and on volunteer healthy controls (HCs) (Table 1). Age and sex-matched HCs were also recruited at the hospital during the same period.

**TABLE 1:**
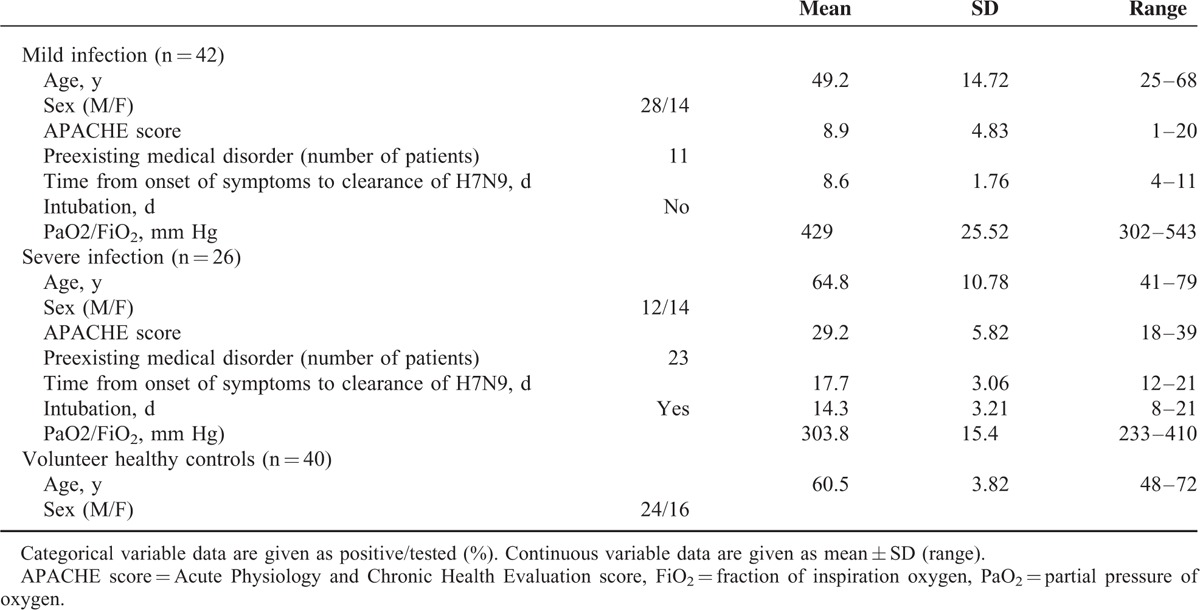
Demographic and Other Background Data

### Flow Cytometry

According to our previous study,^17^ the monoclonal antibodies were as follows: antihuman-PECy5-CD14 (clone RMO52), antihuman-PE-CD16+56/FITC-CD3, antihuman-FITC-CD19 (clone J3–119), antihuman-PerCP-CD3/FITC-CD4/PE-CD8, antihuman-FITC-CD4 (clone 13B8.2), antihuman-APC-CD8 (clone B9.11) (all supplied by Beckman Coulter, CA); antihuman-FITC-HLA-DR (clone Immu-357) (BD Pharmingen, CA); and antihuman-PE-BDCA-1 (Miltenyi, Germany). Intracellular staining with antihuman-PE-IFN-γ (clone 4SB3, eBioscience) was performed using a Cytofix/Cytoperm-Fixation/Permeabilization Kit (BD Pharmingen, CA). Flow cytometry was conducted and analyzed by the FACS Canto II instrument (Becton Dickinson) with Diva 8 software.

### Isolation and Culture of Peripheral Blood Mononuclear Cells

Peripheral blood mononuclear cells (PBMCs) were isolated from samples collected at 1, 3, and 12 months, respectively, after H7N9 infection. Cells were cultured at 2 × 10^6^ cells/mL in RPMI-1640 containing 10% fetal bovine serum (FBS), then stimulated by either 10 μg/mL heat-inactivated H7N9 virus for 8 hours or heat-inactivated *Streptococcus* at 2 × 10^7^ for 24 hours. H7N9 virus and *Streptococcus* were both isolated from the patients.

### Intracellular Cytokine Staining

The PBMCs (1 × 10^6^/mL) were previously stained with anti-CD4-FITC hAb and anti-CD8-APC hAb, and performed by Cytofix/CytopermFixation/Permeabilization Kit after stimuli. Permeabilized cells were incubated with anti-IFN-γ-PE-hAb according to the manufacturer's instructions. Stained cells were washed and the frequency of cytokine-producing CD4^+^ T and CD8^+^ T cells was determined, as we previously described.^24^

### Cytokine and Chemokine Analysis

Tumor necrosis factor (TNF-α), IFN-γ, IFN-α, and IL-6 levels were detected by human enzyme-linked immunosorbent assay (ELISA) Ready-Set-Go Kits (eBioscience). Concentration of the cytokine was expressed as amounts per mL of plasma or supernatant.

The levels of MMPs (Bio-Plex Pro human MMP Panel) were detected by the Luminex enzyme immunoassay (Luminex, TX) and analyzed in vitro.

### Statistical Analyses

Analyses of variance (ANOVAs) and univariate and multivariate Cox analyses were used for comparisons between survivors of mild and severe infection. All data were expressed as means ± standard division (SD), and at least 2 independent experiments were performed. All analysis was performed using GraphPad Prism 6.0 (San Diego, CA). Statistical significance was set at *P* < 0.05.

## RESULTS

### Lymphopenia Had Recovered At 1 Month Postinfection

**FIGURE 1:**
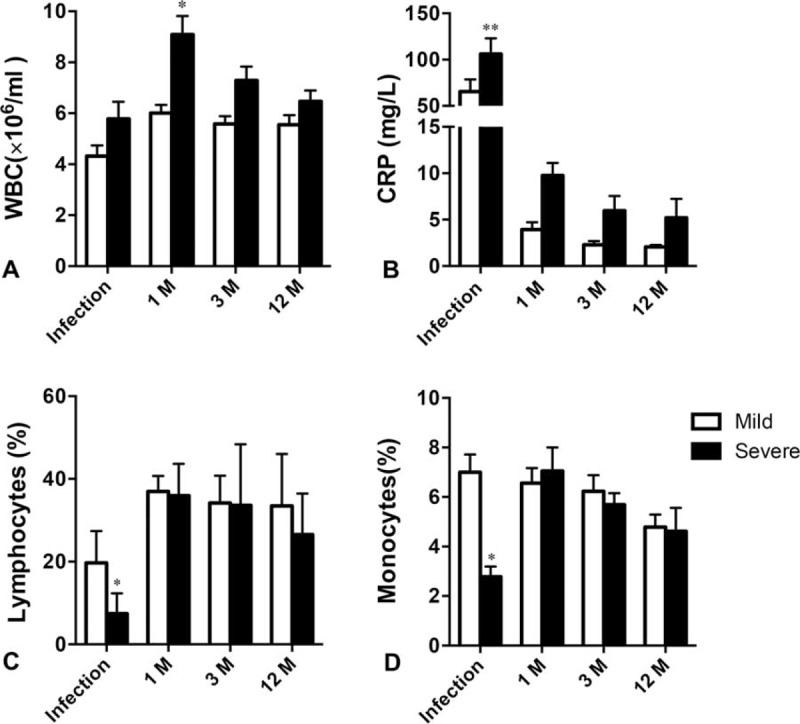
Numbers of white blood cell (WBC) (A), level of C-reactive protein (CRP) (B), lymphocyte (C), and monocyte (D) percentages in survivors of H7N9 infection during follow-ups. Data shown are means ± SD. ^∗^*P* < 0.05, ^∗∗^*P* < 0.01.

To investigate whether the inflammation had subsidized, we followed up the numbers of white blood cells (WBC) and levels of C-reactive protein (CRP). In the whole follow-up, the WBC concentrations of the 2 groups were within the normal limits (4–10 × 10^6^/mL) (Figure 1A). Furthermore, the levels of CRP had both normalized in groups of survivors with mild infection and those who had had severe infection by 1 month postinfection (Figure 1B), though that was higher in patients with severe infection than those with mild infection in infection phage (Figure 1B; *P* = 0.009). The percentages of lymphocyte (Figure 1C) and monocyte (Figure 1D) had normalized by 1 month postinfection. In addition, there were no significant differences in the percentages of lymphocyte and monocyte between either those who had had mild or severe infections or between these groups and HC (*P* = 0.837 and *P* = 0.689, respectively).

### Distribution of Lymphocyte Subsets and Levels of Cytokines in Peripheral Blood Had Normalized At 1 Month Postinfection

**FIGURE 2:**
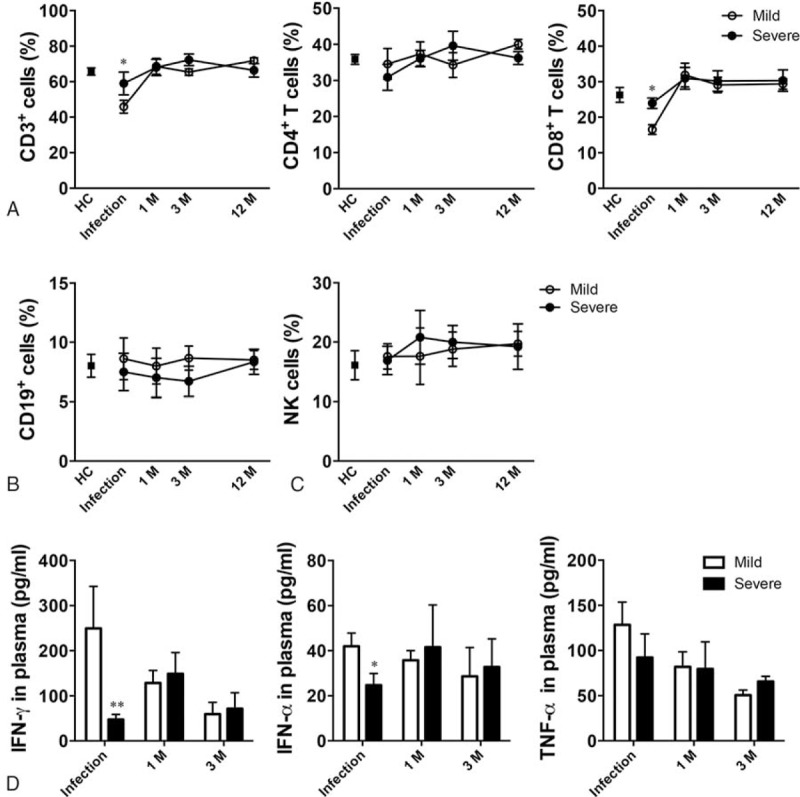
Distribution of lymphocyte subsets and levels of cytokines in peripheral blood in survivors of H7N9 infection. (A) T-cell subgroups; (B) CD19^+^ cells; (C) NK cells; (D) IFN-γ, IFN-α, and TNF-α. Data shown are means ± SD. ^∗^*P* < 0.05, ^∗∗^*P* < 0.01. IFN = interferon, NK = natural killer, TNF = tumor necrosis factor.

There were no significant differences in the percentages of CD4^+^ T cells, B (CD19^+^) cells, and natural killer (NK, CD3^−^, CD16^+^, CD56^+^) cells during the 1-year follow-up between those who had had mild and those who had had severe infection, and between these groups and HC (Figure 2A and B; *P* = 0.463, *P* = 0.535, *P* = 0.291, respectively). There were no significant differences in the CD3^+^ and CD8^+^ T-cell percentages at 1 month postinfection, either between those who had had mild or those who had had severe infection, or between these groups and HC (*P* = 0.252, *P* = 0.762, *P* = 0.458, *P* = 0.771, respectively). This contrasts with our previous finding^17^ that during H7N9 infection, those with severe infection had significantly lower CD3^+^ and CD8^+^ T-cell percentages than those with mild infection.

The plasma levels of the cytokines IFN-γ, IFN-α, and TNF-α during H7N9 infection and at 1 and 3 months postinfection are shown in Figure 2C. During the infection, the levels of IFN-γ and IFN-α were significantly lower in those with severe infection compared with those with mild infection (*P* < 0.001 and *P* = 0.0455). However, at both 1 and 3 months postinfection, there were no significant differences between the levels of these cytokines between those who had had severe and those who had had mild infection, and between these groups and HC (all *P* > 0.05), indicating that the distribution of and the production of cytokines by lymphocytes had returned to normal by 1 month postinfection.

### HLA-DR Expression on CD14-positive Cells Remained Lower Than in HC at 1 Month Postinfection

**FIGURE 3:**
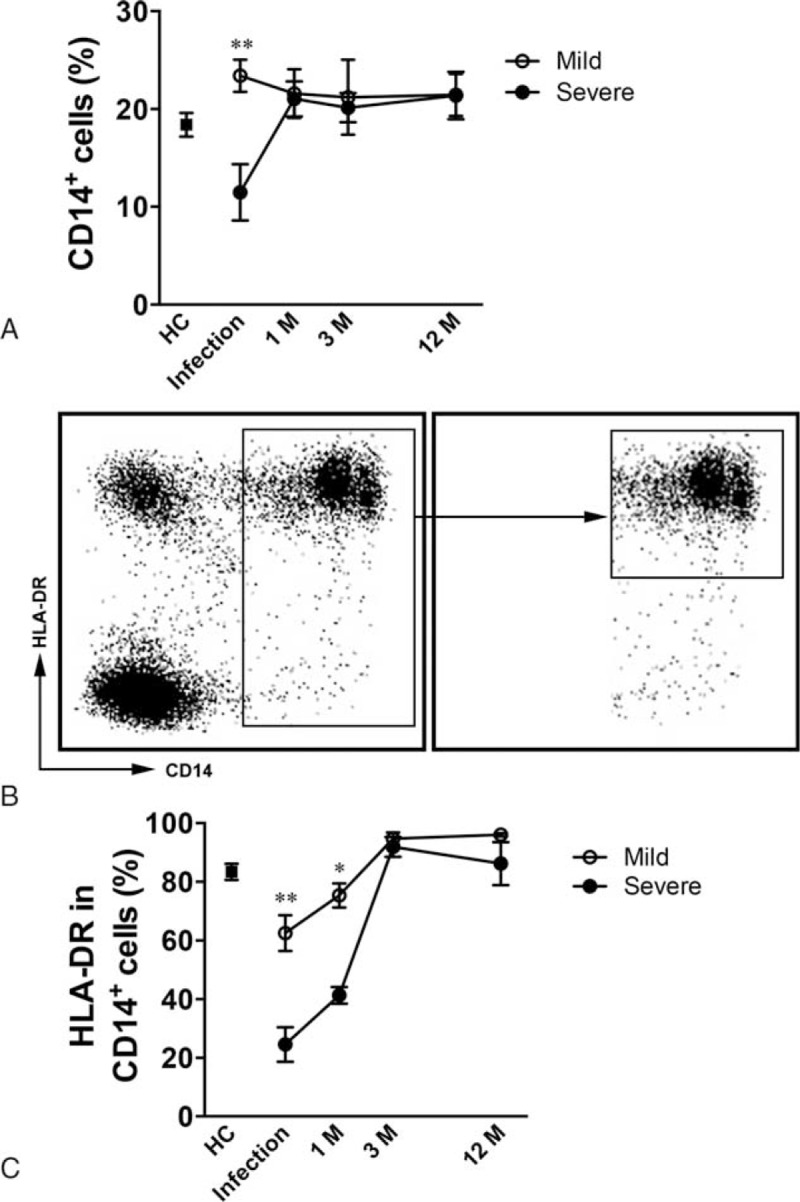
Recovery of monocyte function in survivors of H7N9 infection. (A) CD14^+^ cell percentages. Representative images (B) and statistical data (C) of HLA-DR expression on CD14^+^ cells. Data shown are means ± SD. ^∗^*P* < 0.05, ^∗∗^*P* < 0.01.

The proportion of CD14^+^ cells was significantly higher at 1 month postinfection in those who had had severe infection when compared with the proportion during H7N9 infection (Figure 3A; *P* = 0.013). This is consistent with the significant increase in the monocyte percentage at follow-up after infection compared with during infection, as CD14 is mainly expressed on monocytes.

The expression of HLA-DR on CD14^+^ cells was significantly higher postinfection compared with during infection in those who had had mild infection and was similar to that in HCs (*P* = 0.005 and *P* = 0.756). Although the expression of HLA-DR on CD14^+^ cells was higher during follow-up compared with during infection in those who had had severe infection (*P* = 0.034), it was still significantly lower at 1 month postinfection compared with that in those who had had mild infection (*P* < 0.001). At 3 months postinfection, there was no significant difference in the expression of HLA-DR on CD14^+^ cells in those who had had severe infection compared with those who had had mild infection (*P* = 0.648) (Figure 3B and C). There was no significant difference in HLA-DR expression on dendritic cells (DCs) in either those who had had mild or those who had had severe infection during the 1-year follow-up period (Supplementary Figure 1).

These results imply that at 1 month postinfection, antigen presentation remained impaired in those who had had severe H7N9 infection, but not in those who had had mild infection.

### Antigen Presentation After Exogenous Stimulation Was Impaired At 1 Month Postinfection in Those Who Had Had Severe H7N9 Infection

**FIGURE 4:**
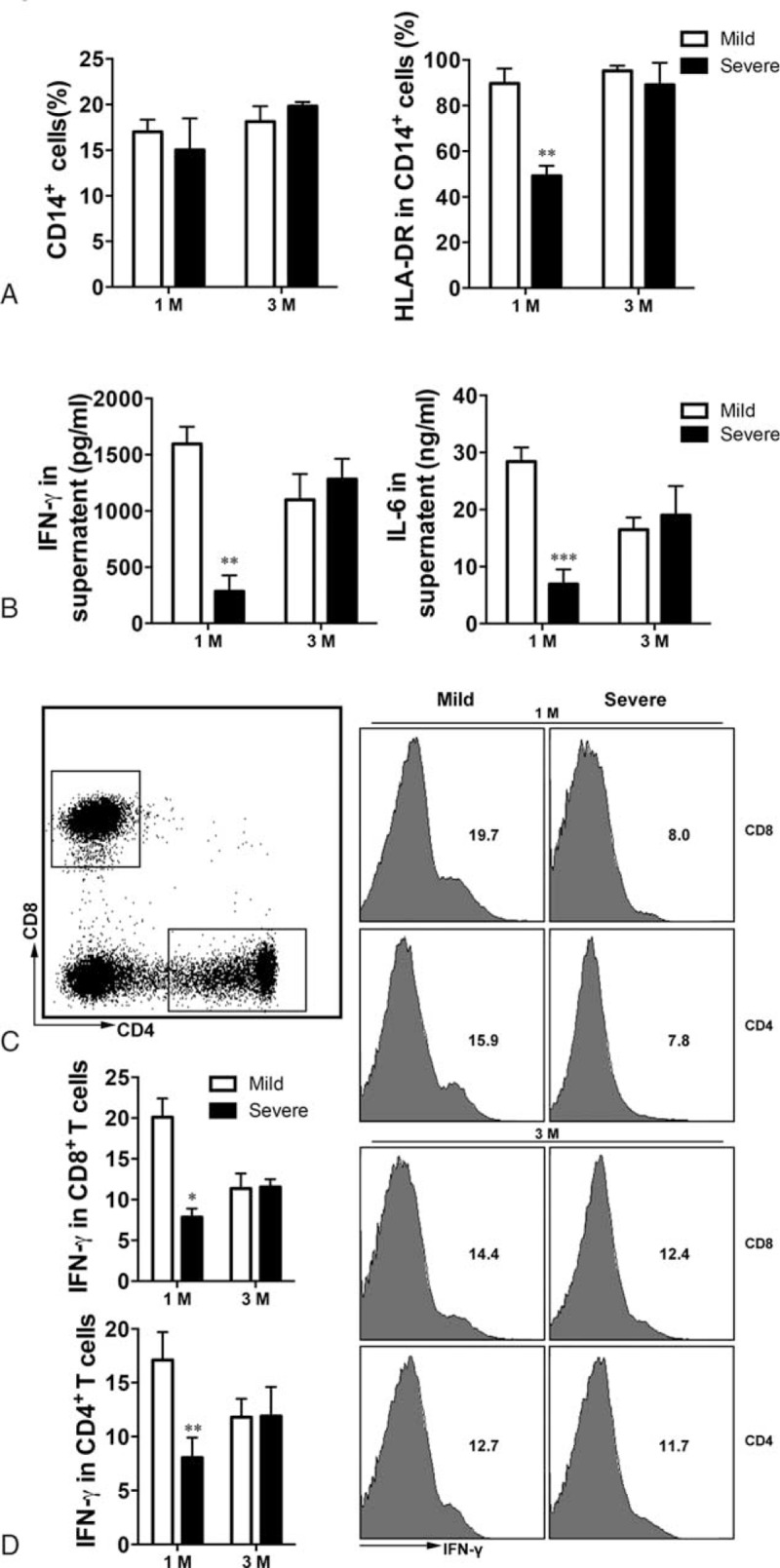
The antigen-presentation capability of survivors of H7N9 infection. (A) CD14^+^ proportion and HLA-DR expression on CD14^+^ cells detected by flow cytometry after *Streptococcus* stimulation. (B) IFN-γ and IL-6 levels in the supernatant measured by ELISA after *Streptococcus* stimulation. (C) Intracellular IFN-γ production by CD4^+^ and CD8^+^ T cells detected by flow cytometry after *Streptococcus* stimulation. Also, statistical data were shown in (D). Data shown are means ± SD. ^∗^*P* < 0.05, ^∗∗^*P* < 0.01. ELISA = enzyme-linked immunosorbent assay, IFN = interferon, IL-6 = interleukin-6.

The expression of HLA-DR on CD14^+^ cells was significantly higher after *Streptococcus* stimulation in PBMCs, though there was no difference in CD14^+^ cells in those who had had mild H7N9 infection compared with those who had had severe infection (Figure 4A; *P* = 0.003 and *P* = 0.273). IFN-γ and IL-6 levels in the culture supernatant from PBMCs after *Streptococcus* stimulation were significantly higher at 1 month postinfection in those who had had mild infection compared with those who had had severe infection (Figure 4B; both *P* < 0.001). At 3 months postinfection, there was no significant differences in HLA-DR expression on CD14^+^ cells and IFN-γ and IL-6 levels in the culture supernatant between those who had had mild and those who had had severe infection (Figure 4A and B; *P* = 0.493 and *P* = 0.412).

Interferon-γ production, measured by flow cytometry, from CD4^+^ and CD8^+^ T cells after *Streptococcus* stimulation, was significantly higher in those who had had mild infection and also those who had had severe infection at 1 month postinfection. This is consistent with the results we obtained regarding antigen presentation capability (Figure 4C and D; *P* = 0.006 and *P* = 0.047). At 3 months postinfection, there was no significant difference in IFN-γ production between those who had had mild and those who had had severe infection (Figure 4C and D; *P* = 0.935 and *P* = 0.976).

Surprisingly, at 1 month postinfection, there were no significant difference in the expression of HLA-DR on CD14^+^ cells or IFN-γ production from CD4^+^ T cells in those who had had mild infection compared with those who had had severe infection, after stimulation with inactive H7N9 virus when compared with no stimulation (Supplement Figure S2; *P* = 0.791 and *P* = 0.697).

### There May Be Tissue Remodeling At 1 Month Postinfection In Those Who Had Had Severe H7N9 Infection

**FIGURE 5:**
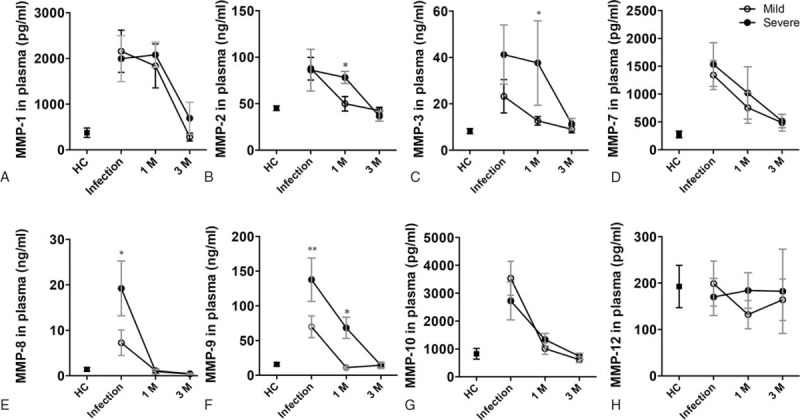
MMP plasma levels (A–H) in survivors of H7N9 infection. Data shown are means ± SD. ^∗^*P* < 0.05, ^∗∗^*P* < 0.01. MMP = matrix metalloproteinase.

The MMP family plays an important role in tissue remodeling associated with various physiological or pathological processes.^19,20^ Excess MMPs degrade the structural proteins of the aortic wall, which represented increased possibility of tissue injury and remodeling. We found that the plasma levels of MMP1, MMP2, MMP3, MMP7, MMP8, MMP9, and MMP10 were higher in patients during H7N9 infection than in HCs, and that the plasma levels of MMP8 and MMP9 were significantly higher in those with severe infection compared with those with mild infection (*P* = 0.005 and 0.052). At 1 month postinfection, MMP plasma levels in those who had had mild infection were significantly lower than they had been during H7N9 infection and were similar to the levels in HC. In contrast, at 1 month postinfection, the plasma levels of MMP2, MMP3, and MMP9 in those who had had severe infection remained significantly higher than in those who had had mild infection (*P* = 0.023, *P* = 0.048, *P* = 0.032, respectively). At 3 months postinfection, the plasma levels of these MMPs were similar in those who had had mild and also those who had had severe infection (Figure 5).

As the MMP family plays an important role in regulating tissue remodeling, these results suggest that there may be significant tissue remodeling at 1 month postinfection after severe H7N9 infection.

## DISCUSSION

To date, 3 waves of H7N9 virus infection have affected eastern and southern China. The infection usually causes a severe illness, and although some patients recover, the mortality is high.^21,22^

We previously described the immunological responses seen during H7N9 infection, comparing them in those with mild and severe infection. Severe H7N9 infection is characterized by lymphopenia, lower antigen-presenting capability, and impaired T-cell responses. In addition, the viral load in infection phage was higher and the duration of infection was longer in patients with severe infection when compared with those with mild infection; this might affect the clinical outcome. We speculated that the duration of immunological impairment, with associated persistence of the virus, might arise as a consequence of a compromised ability to eliminate the virus.^17,23^

In the current study, we investigated the recovery in immunological function during a 1-year follow-up period in patients surviving mild and severe H7N9 infection. By 1 month postinfection, the peripheral lymphocyte and monocyte percentages were similar to those in uninfected individuals, and total T and B lymphocyte and NK-cell percentages, and also CD3^+^ and CD8^+^ T-cell percentages were similar in those who had had mild and those who had had severe infection. Moreover, plasma levels of IFN-γ, IFN-α, and TNF-α in survivors of mild and severe infection were similar. Therefore, it can be said that the distribution of lymphocytes and the general levels of cytokines in survivors of H7N9 infection return quickly to normal.

However, at 1 month postinfection, HLA-DR expression on monocytes from survivors who had had severe infection was low compared with that from survivors who had had mild infection, although the expression was similar in both groups at 3 months postinfection. HLA-DR expression on DCs was similar in both groups throughout the follow-up period despite the role in antigen-presenting.^24^ Overall, our findings indicated that severe H7N9 infection is associated with persistent impairment in the antigen presenting capability of monocytes.

Further evidence of the impaired antigen-presenting capability of monocytes persisting at 1 month after severe H7N9 infection came both from our studies in which PBMCs were stimulated with heat-inactivated *Streptococcus,* and from our studies of T-cell responses after *Streptococcus* stimulation. IL-6 and IFN-γ levels in the supernatant were lower in those who had had severe infection compared with those who had had mild infection. In addition, IFN-γ production by CD4^+^ and CD8^+^ T cells was higher 1 month postinfection in those who had had mild infection compared with those who had had severe infection. However, stimulation with inactive H7N9 virus did not activate monocytes and T-cell responses, mainly due to the tolerated T cells exposed to H7N9 virus for long. In a study of recovery of other respiratory diseases, a dramatically reduced response to serum antibody in survivors of H5N1 virus infection has also been reported.^25^ The lower antigen-presenting capability of monocytes with weaker T-cell responses in survivors of severe infection with H7N9 virus is likely to cause an increased susceptibility to secondary bacterial infections during recovery from the initial infection.

Increased plasma levels of MMPs can indicate tissue remodeling. MMP2 and MMP9 are crucial in the hydrolysis of fibronectin and collagens IV. MMP1, MMP3, MMP8, MMP10, and MMP12 are mainly involved in the hydrolysis of collagens (I, II, III).^26,27^ MMP1, MMP3, and MMP12 contribute to hydrolysis of MMP9 and pro-MMP9.^28^ MMP2 is an interstitial collagenase.^29^ MMP7 is closely involved with transferrin and casein hydrolysis.^30^ And our previous study inferred MMP7 could play an important role in influenza infection.^31^ At 1 month postinfection, we found higher plasma levels of MMP2, MMP3, and MMP9 in survivors of severe H7N9 infection. It is possible that ongoing tissue remodeling adds to the feeble recovery seen after severe H7N9 infection.^32,33^

In our previous study, treatment with glucocorticoid induced no significant difference between 2 groups of H7N9 patients. Besides, all patients were given oseltamivir, and the time span between onset of symptoms and initiation of this drug showed no difference between patients of mild and severe infection. Comorbidities of the patients in acute phage mainly manifest as pneumonia, ARDS, and acute kidney injury. Incidence of pneumonia showed no difference between the 2 groups of survivors, whereas ARDS and acute kidney injury were rare in survivors. Even so, low probability of ARDS and acute kidney injury might influence the frequency of CD14^+^ cells.

The higher mean age in survivors with severe infection than those with mild infection suggested that the age is a risk factor, which could influence the results caused by H7N9 infection. In our previous study, though the frequency of CD14^+^ cell and HLA-DR expression on CD14^+^ cells showed no significant difference between the age-matched healthy individuals in both the groups.^17^ However, infection with H7N9 may induce the down-regulation of the frequency of CD14^+^ cells and the HLA-DR expression on CD14^+^ cells in older patients. We speculate that the recovery of H7N9 infection could also be influenced by the potentially lower immune response in older patients.

However, we followed up the numbers of WBC and levels of CRP, which mainly represent the possibility of infection. We found that there were higher levels of WBC and CRP in survivors who had had severe infection (close to upper normal limit) than those who had had mild infection at 1-month follow-up, though the numbers of WBC showed no difference between the 2 groups in acute phage, and levels of CRP in all patients had normalized in 1-month follow-up. (Figure 1A and B; *P* = 0.021 and *P* = 0.05). All these data indicated survivors of severe infection were more susceptible to secondary bacterial infection than survivors of mild infection.

In conclusion, after severe H7N9 virus infection, immune recovery is delayed in those with severe infection compared with those with mild infection. This is a consequence of an impaired antigen-presenting ability of monocytes, leading to impaired T-cell responses. This may cause an increased susceptibility to secondary bacterial infections. It illustrates why patients with severe infection are more likely to be exposed to secondary bacterial infections, which is one of the important deadly reasons. These findings may benefit our laboratory studies and clinical diagnosis and treatment on infection with avian influenza. Enhancement of antigen-presenting capability may be conducive not only to infection defense but also to development of immune treatment.

## Supplementary Material

Supplemental Digital Content
