# Supplementary material for: Severe Infection With Avian Influenza A Virus is Associated With Delayed Immune Recovery in Survivors
Source: Medicine (Baltimore). 2016 Feb 8;95(5):e2606. doi: 10.1097/MD.0000000000002606 (PMC4748887; doi:10.1097/MD.0000000000002606)
Supplement: Supplemental Digital Content [file medi-95-e2606-s001.pdf]

Severe infection with Avian influenza A virus is associated with delayed immune recovery in survivors

Jianing Chen, Guangying Cui, Chong Lu, Yulong Ding, Hainv Gao, Yixin Zhu, Yingfeng Wei, Lin Wang, Toshimitsu Uede, Lanjuan Li & Hongyan Diao

Supplementary Figure 1

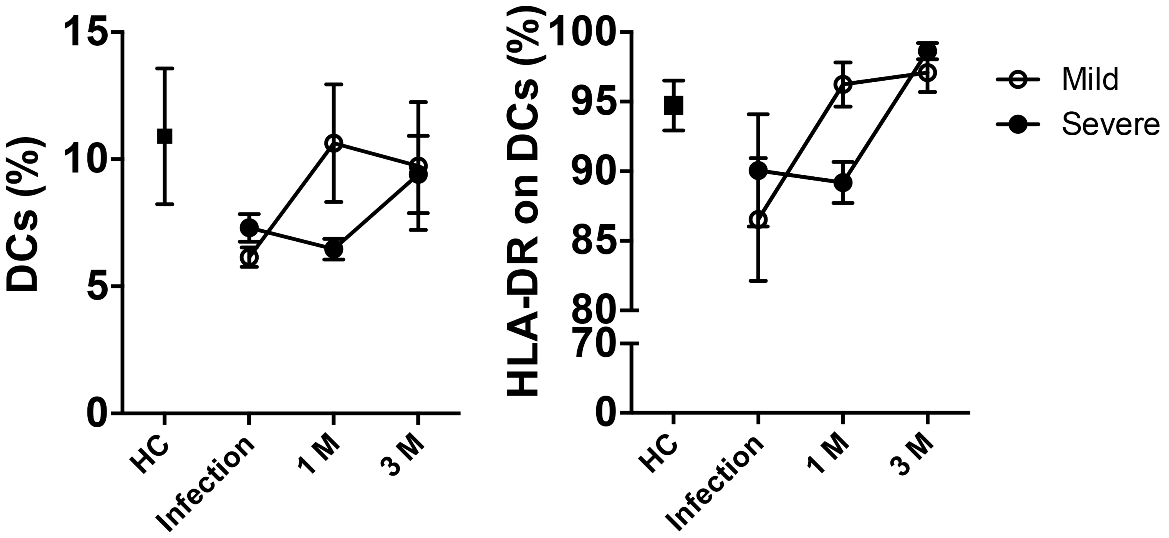

Dendritic cells (DCs) percentages and HLA-DR expression, determined by flow cytometry, on DCs during and after infection with H7N9.

Severe infection with Avian influenza A virus is associated with delayed immune recovery in survivors

Jianing Chen, Guangying Cui, Chong Lu, Yulong Ding, Hainv Gao, Yixin Zhu, Yingfeng Wei, Lin Wang, Toshimitsu Uede, Lanjuan Li & Hongyan Diao

Supplementary Figure 2

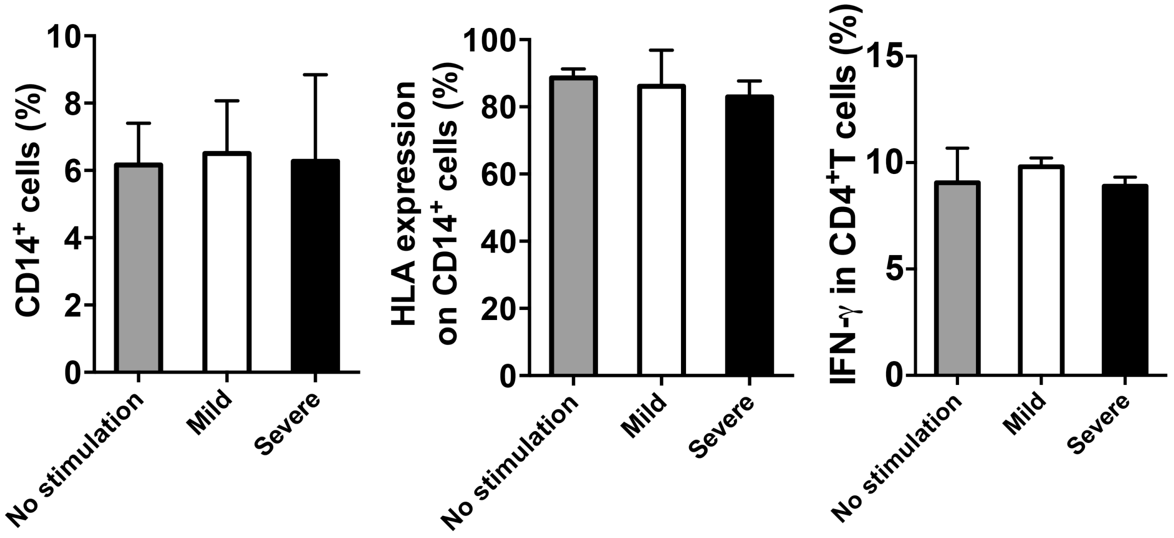

Effects of PBMCs stimulation with or without heat-inactivated H7N9 virus.

Shown are $CD14^+$ cells percentages, HLA expression on $CD14^+$ cells and IFN-γ production by $CD4^+$ T cells measured by flow cytometry.
